# Feedback-Driven Mechanisms Between Phosphorylated Caveolin-1 and Contractile Actin Assemblies Instruct Persistent Cell Migration

**DOI:** 10.3389/fcell.2021.665919

**Published:** 2021-04-12

**Authors:** Xuemeng Shi, Zeyu Wen, Yajun Wang, Yan-Jun Liu, Kun Shi, Yaming Jiu

**Affiliations:** ^1^The Joint Program in Infection and Immunity, Guangzhou Women and Children’s Medical Center, Guangzhou Medical University, Guangzhou, China; ^2^The Joint Program in Infection and Immunity, Institut Pasteur of Shanghai, Chinese Academy of Sciences, Shanghai, China; ^3^Key Laboratory of Molecular Virology and Immunology, The Center for Microbes, Development and Health, Institut Pasteur of Shanghai, Chinese Academy of Sciences, Shanghai, China; ^4^University of Chinese Academy of Sciences, Beijing, China; ^5^Shanghai Institute of Cardiovascular Diseases, and Institutes of Biomedical Sciences, Zhongshan Hospital, Fudan University, Shanghai, China

**Keywords:** actin filaments, actomyosin bundles, Arp2/3 complex-dependent lamellipodia, directional cell migration, caveolin-1

## Abstract

The actin cytoskeleton and membrane-associated caveolae contribute to active processes, such as cell morphogenesis and motility. How these two systems interact and control directional cell migration is an outstanding question but remains understudied. Here we identified a negative feedback between contractile actin assemblies and phosphorylated caveolin-1 (CAV-1) in migrating cells. Cytoplasmic CAV-1 vesicles display actin-associated motilities by sliding along actin filaments or/and coupling to do retrograde flow with actomyosin bundles. Inhibition of contractile stress fibers, but not Arp2/3-dependent branched actin filaments, diminished the phosphorylation of CAV-1 on site Tyr14, and resulted in substantially increased size and decreased motility of cytoplasmic CAV-1 vesicles. Reciprocally, both the CAV-1 phospho-deficient mutation on site Tyr14 and CAV-1 knockout resulted in dramatic AMPK phosphorylation, further causing reduced active level of RhoA-myosin II and increased active level of Rac1-PAK1-Cofilin, consequently led to disordered contractile stress fibers and prominent lamellipodia. As a result, cells displayed depolarized morphology and compromised directional migration. Collectively, we propose a model in which feedback-driven regulation between actin and CAV-1 instructs persistent cell migration.

## Introduction

The actin cytoskeleton and caveolae are important players in regulating cell morphology and directional migration. Early electron microscopy studies revealed the association of caveolae with filamentous actin (Rohlich and Allison, 1976; Rothberg et al., 1992; Valentich et al., 1997), and later the actin-binding protein filamin A was found to be important for caveolae co-alignment with actin stress fibers (Muriel et al., 2011). However, aside from membrane-located caveolae which has been extensively studied as tension sensor and intimately interact with cortical actin (Sinha et al., 2011), the interactive mechanisms between actin and cytoplasmic caveolin-1 (CAV-1), principal structural component of caveolae (Navarro et al., 2004), have remained elusive. For example, how specific actin assemblies control the cytoplasmic CAV-1, and reciprocally, how CAV-1 protein *per se* regulate signaling cascades to control dynamic actin assemblies and subsequent cell behavior.

Cytoplasmic CAV-1 could either form pivotal structural component for caveolae, or form non-caveolae CAV-1 vesicles/foci (Razani et al., 2002b; Pol et al., 2020). It is speculated, but no direct evidence yet, that the organization and trafficking of both CAV-1 forms are tightly associated with distinct intracellular actin assemblies and critical actin-binding proteins. Global disruption of the actin network by cytochalasin D treatment was found to form strong clustering of CAV-1 (Fujimoto et al., 1995; Mundy et al., 2002). Patches of non-polymerized actin are concentrated around rapidly moving CAV-1 spots (Echarri et al., 2012; Stoeber et al., 2012). Depletion of myosin-1c, one actin associated motor protein, induces a perinuclear accumulation of CAV-1 and a decrease in caveolar density (Hernandez et al., 2013). Knockdown of actin nucleator mDia1 and Abl kinase induces caveolae clustering, whereas active mDia1 increases stress fiber formation, and reduces the numbers of clustered “caveolar rosettes” superstructures (Echarri et al., 2012). Nevertheless, silencing the Arp2/3 complex or its activators N-WASP and cortactin does not affect the endocytic process of CAV-1 vesicles from the plasma membrane (Echarri et al., 2012), but how their roles during the dynamic regulation of intracellular CAV-1 remain less examined.

Actin assemblies require the spatial and temporal integration of different signaling components (Ridley et al., 2003; Vicente-Manzanares et al., 2005). By coordinating the active levels of Rho family of small GTPases and ultimately, actin polymerization, Rac1 regulates lamellipodia protrusion and membrane ruffles and Cdc42 triggers filopodia, whereas RhoA regulates the formation of actomyosin-formed stress fibers and cell contractility (Ridley et al., 2003; Raftopoulou and Hall, 2004; Vicente-Manzanares et al., 2005).

Previous reports suggest that CAV-1 promotes cell migration (Zhang et al., 2000; Gonzalez et al., 2004; Grande-Garcia et al., 2007; Joshi et al., 2008; Hill et al., 2012). For example, CAV-1 depletion in mouse embryonic fibroblasts has been shown to elevate the active level of Rac1 and Cdc42 while decrease the RhoA activity, thus lead to defects in actin remolding associated cell motility (Grande-Garcia et al., 2007). However, other studies indicate that CAV-1 could be a negative regulator in the context of cell migration. For instance, restoration of CAV-1 expression in MTLn3 cells reduces the chemotactic directed cell migration (Zhang et al., 2000). Likewise, CAV-1 knockdown increased the persistent migration toward sphingosine-1 phosphate in bovine aortic endothelial cells (Gonzalez et al., 2004). Although some of these discrepancies could be ascribed to technical or cell type specificity issues, it appears important to ascertain what kind of role, if any, CAV-1 plays in the coordinated processes of directional migration through controlling of actin assembly and remodeling.

In this study we identified that the cytoplasmic CAV-1 vesicles move concurrently with the retrograde flow of actin filaments toward the deep cytoplasmic perinuclei region, and meanwhile are also able to laterally slide along the actin filaments. Moreover, contractile actomyosin bundles determine the organization and dynamics of intracellular CAV-1 by disturbing its phosphorylation level, whereas branched actin structures are dispensable. Importantly, CAV-1 depletion leads to compromised intrinsic persistent migration through remolding the active level of AMPK, which in turn regulates the activity of Rac1 and downstream PAK1 and Cofilin for protrusive lamellipodia, and correspondingly the activity of RhoA and subsequent changes in stress fibers formation and contraction. Furthermore, the phosphorylation of CAV-1 on site Tyr14 plays an essential role in regulating these kinase-GTPase signaling axis governed actin remodeling. Taken together, our study comprehensively investigates and hence provides new insights into the interactive feedback mechanisms between actin assemblies and cytoplasmic CAV-1 in the context of cell migration.

## Results

### Cytoplasmic CAV-1 Interacts With Actin Filaments in Human Osteosarcoma Cells

**FIGURE 1:**
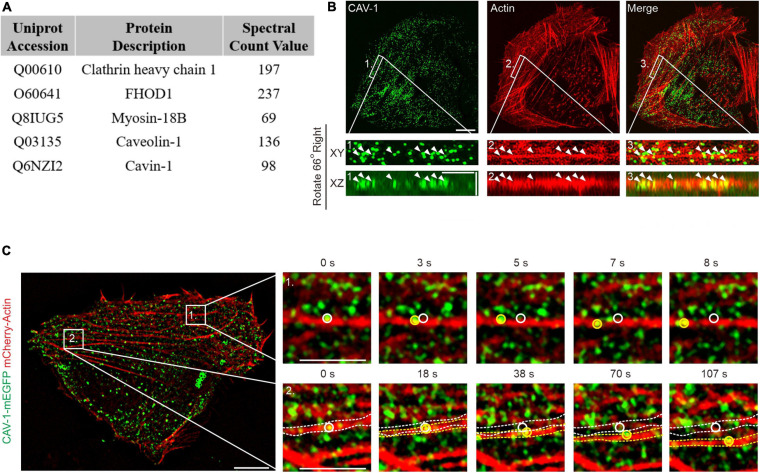
The association of actin filaments with cytoplasmic CAV-1 in human osteosarcoma cells. **(A)** Top protein hits from the BioID screen for identification of Tpm3.1-associated proteins in U2OS cells. **(B)** Localization of endogenous CAV-1 and actin filaments in U2OS cells detected by CAV-1 antibody and fiuorescent phalloidin, respectively. Magnified regions from the area indicated by white boxes demonstrate that cytoplasmic CAV-1 aligns with actin filaments (white arrowheads). The bottom panels show the orthographic view of the enlarged region, where “XY” and “XZ” indicated different cross-sections. Bars, 10 μm (in cell images), 2 μm (in magnified images and orthographic views). **(C)** Time-lapse imaging of U2OS cells co-expressing CAV-1-mEGFP and mCherry-actin revealing that actin-associated cytoplasmic CAV-1 vesicles move along actin filaments (Box 1) and do retrograde flow with contractile arcs (Box 2). The magnified regions of Box 1 and 2 in different time point are shown on the right panels, respectively. White and yellow circles and dotted lines in the magnified regions indicate the starting and ending position of discrete CAV-1 tagged vesicles and contractile arcs, respectively. The recording was set as every 1 s for 200 s. Bars, 10 μm (in cell image) and 5 μm (in Box 1 and Box 2).

To identify novel actin filament interactions, we performed a proximity-dependent biotin identification (BioID) analysis (Roux et al., 2012) on human osteosarcoma cells (U2OS) using a biotin ligase fused to Tropomyosin-3.1 (Tpm3.1), a central actin filaments component (Supplementary Figure 1A; Parreno et al., 2020). Among the high-confidence interactors, CAV-1, which has been used as a measure of cytoplasmic trafficking of both individual and clustered forms of caveolae (Mundy et al., 2002; Echarri et al., 2012), was chosen for further investigation (Figure 1A).

Previous studies have demonstrated co-alignment of CAV-1 with filamentous actin in a number of cell types, such as NIH3T3 cells, epithelial cells, fibroblasts, myofibroblasts and muscle cells (Rohlich and Allison, 1976; Rothberg et al., 1992; Valentich et al., 1997). Immunofluorescence imaging on U2OS cells, which are much adherent and show clear endogenous staining of cytoskeleton (Jiu et al., 2015, 2019), revealed an extensive actin network and readily recognizable CAV-1 vesicles, and the cytoplasmic CAV-1 vesicles were found aligned with actin filaments occasionally (Figure 1B), indicating the enability to determine the association between these structures in U2OS cells.

We next assess the actin associated intracellular movement of CAV-1, using expression of fluorescent protein fusion constructs. This revealed a punctate pattern that colocalized with endogenous CAV-1 but showed no co-distribution with Rab8-labeled endosomes, indicating a proper distribution with respect to the endogenous protein (Supplementary Figure 1B). By using wheat germ agglutinin (WGA), a small-molecule marked the plasma membrane, we showed that the actin filaments-associated CAV-1 signals were mainly localized in the cytoplasm (Supplementary Figure 1C). To record the cytoplasmic CAV-1 vesicles, we optimized the imaging focal plane based on the clear and sharp appearance of DAPI-labeled nucleus in the following experiments, which demarcated the cytoplasmic (middle layer) rather than the plasma membrane (bottom layer) field (Supplementary Figure 1D).

Live cell imaging enabled us to identify that while the CAV-1 vesicles at the perinuclear area did not exhibit regular movements, others which close to cell periphery associated with contractile actin transverse arcs and displayed retrograde flow toward the cell center, accompanying with cases of sliding along actin filaments (Figure 1C and Supplementary Videos 1, 2). Together, these data provide direct evidences that CAV-1 vesicles interact with actin network in human osteosarcoma cells.

### Contractile Actin Assemblies Are Critical for the Organization and Dynamics of CAV-1 Vesicles by Disturbing Its Phosphorylation Level

**FIGURE 2:**
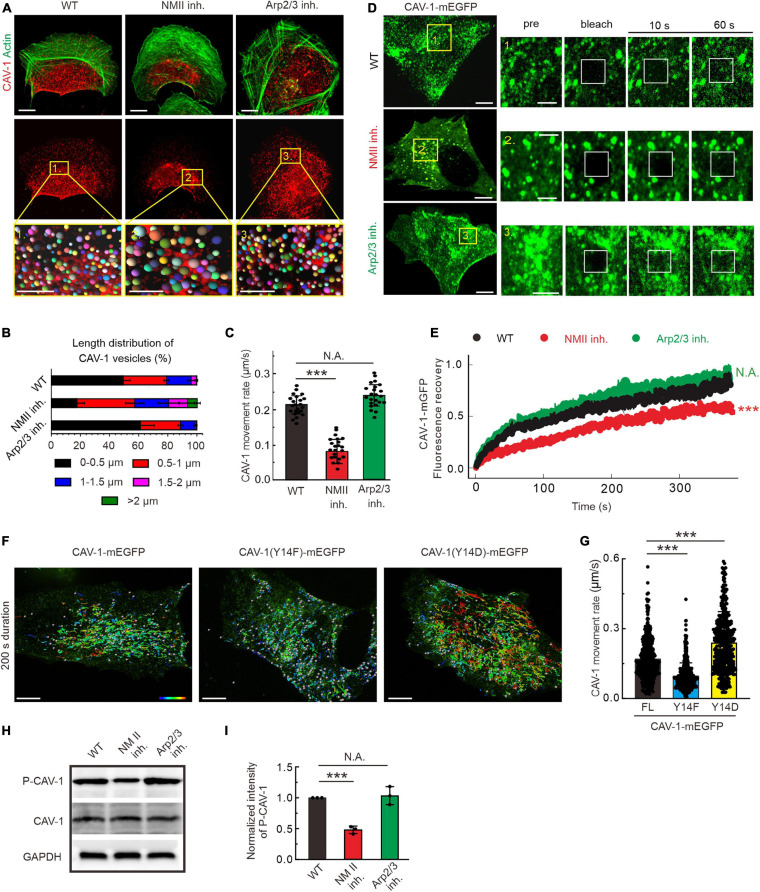
The organization, dynamics and phosphorylation of CAV-1 are regulated by contractile actin assemblies. **(A)** Immunofluorescence staining of endogenous F-actin and CAV-1 vesicles in wild type (WT), myosin II inhibition (NMII inh.) and Arp2/3 complex inhibition (Arp2/3 inh.) cells, respectively. The representative analysis of CAV-1 positive dots detected by Imaris of magnified yellow boxes 1, 2, and 3 on the top panels are marked as balls which are randomly colored on the lower panels. The size of the color balls indicates the calculated sizes of CAV-1 vesicles. Bars, 10 μm (in cell images) and 5 μm (in the magnified box). **(B)** The length distribution of CAV-1 vesicles. The number of vesicles in each group of size is divided by the total CAV-1 number of the same cell. *n* = 25,602 vesicles from 32 WT cells, 13,456 vesicles from 31 myosin II inhibition cells, and 26,103 vesicles from 29 Arp2/3 complex inhibition cells. **(C)** Quantification of the movement rate of CAV-1 vesicles in wild-type (*n* = 26), myosin II inhibition (*n* = 22), and Arp2/3 complex inhibition (*n* = 23) cells. **(D)** FRAP analysis of CAV-1-mGFP dynamics in WT, NM II inh. and Arp2/3 inh. cells. Magnified regions represent time-lapse images of the bleached regions. Bars, 10 μm (in cell images) and 5 μm (in the magnified time-lapse image). **(E)** Normalized average FRAP recovery curves of CAV-1-mEGFP in WT (*n* = 23), NM II inh. (*n* = 22), and Arp2/3 inh. (*n* = 22) cells. **(F)** The representative 200 s duration dot tracking analysis of mEGFP tagged CAV-1, CAV-1(Y14F) and CAV-1(Y14D) vesicles in CAV-1 KO cells by Imaris. Color-coded bar from blue to red indicates the tracked mean speeds ranging from 0 to 0.4 μm/s. Bars, 10 μm. **(G)** Quantification of the movement rate of CAV-1 positive vesicles. *n* = 419/496/432 vesicles from 10 CAV-1/CAV-1(Y14F)/CAV-1(Y14D)-mEGFP expressing cells. **(H,I)** Western blot analysis **(H)** and quantifications **(I)** of phosphorylated CAV-1 (Tyr14) (compared to total CAV-1) in WT, NM II inh. and Arp2/3 inh. cell lysates. The obtained intensity value from wild-type cells was set to 1. *n* = 3. Data in panel **(C,G,I)** are presented as mean ± SD. ***P ≤ 0.001; N.A., not significant (one-way ANOVA). All the data are from three independent experiments.

Global pharmacological disruption of actin cytoskeleton in U2OS cells leads to an increase of cytoplasmic CAV-1 clustering (Supplementary Figures 2A,B). In migrating cells actin filaments are able to assemble into distinct three-dimensional structures. To further elucidate the underlying regulation by these specific actin arrangements to cytoplasmic CAV-1 vesicles, we employed different actin-directed drugs with specific targets (Figure 2A). Blebbistatin and CK666 inhibiting myosin II ATPase activity and Arp2/3 complex were applied, and thereby interfering with the formation of contractile and branched filament assemblies, respectively (Kovacs et al., 2004; Chanez-Paredes et al., 2019). Neither of the drugs affected the transcription and expression level of CAV-1 (Supplementary Figures 2C,D), while fluorescence imaging witnessed their expected effects on actin organization with extensive loss of prominent contractile bundles after exposure to blebbistatin and of lamellipodia after CK666 treatment, respectively (Figure 2A). Enlarged CAV-1 vesicles appeared upon blebbistatin addition, but remain similar size as in wild-type under CK666 circumstance (Figure 2B). Moreover, the motility of cytoplasmic CAV-1 vesicles was significantly compromised when myosin II activity, but not Arp2/3, was inhibited (Figure 2C). Having identified that actomyosin dependent contractility is important for cytoplasmic CAV-1, prompted us to confirm this influence by using alternative approach. Myosin-18B depletion cells are reluctant to form large myosin II stack and subsequent result in less contractile actin stress fibers (Jiu et al., 2019), while elimination of ARPC2, component of Arp2/3 complex (Goley and Welch, 2006), leads to less branched actin formed lamellipodia. Consistent with the results by pharmacological inhibitors, increased size and decreased motility of CAV-1 vesicles were observed in myosin-18B knockout cells, but not ARPC2 knockdown cells (Supplementary Figures 2E–H). Fluorescence recovery after photobleaching (FRAP) measurement was also applied to validate the relatively slow kinetics of cytoplasmic CAV-1 molecules lacking of contractile actomyosin bundles (Figures 2D,E).

Tyr14 is an important phosphorylation site to modulate CAV-1 activity and subsequent focal adhesion dynamics, cell migration and mechanical stimuli (Navarro et al., 2004; Zimnicka et al., 2016; Wong et al., 2020). The phospho-deficient (Y14F) and phospho-mimic (Y14D) mutants were exogenously expressed in CAV-1 knockout (CAV-1 KO) cells, respectively, to eliminate the interference of endogenous CAV-1. With tracking of real-time imaging, we revealed that the CAV-1 (Y14D) vesicles represented faster motility while the CAV-1 (Y14F) vesicles are overall slower than wild-type (Figures 2F,G), suggesting that CAV-1 phosphorylation is intimately correlates with its cytoplasmic dynamics.

Notably in this context, we examined the effects of above drugs on the phosphorylation state of CAV-1. Western blot indicated that the phosphorylated CAV-1 level on site Tyr14 was apparently decreased in actomyosin inhibition vs. wild-type, but was not significantly altered upon Arp2/3 inhibition (Figures 2H,I). Taken together, these data demonstrate that contractile actin assemblies are critical for the organization and dynamics of cytoplasmic CAV-1, most likely by regulating its phospho-related active level.

### Phospho-Deficient and Depletion of CAV-1 Compromises the Assembly of Contractile Stress Fibers by Deactivating RhoA-Dependent Myosin Phosphorylation

**FIGURE 3:**
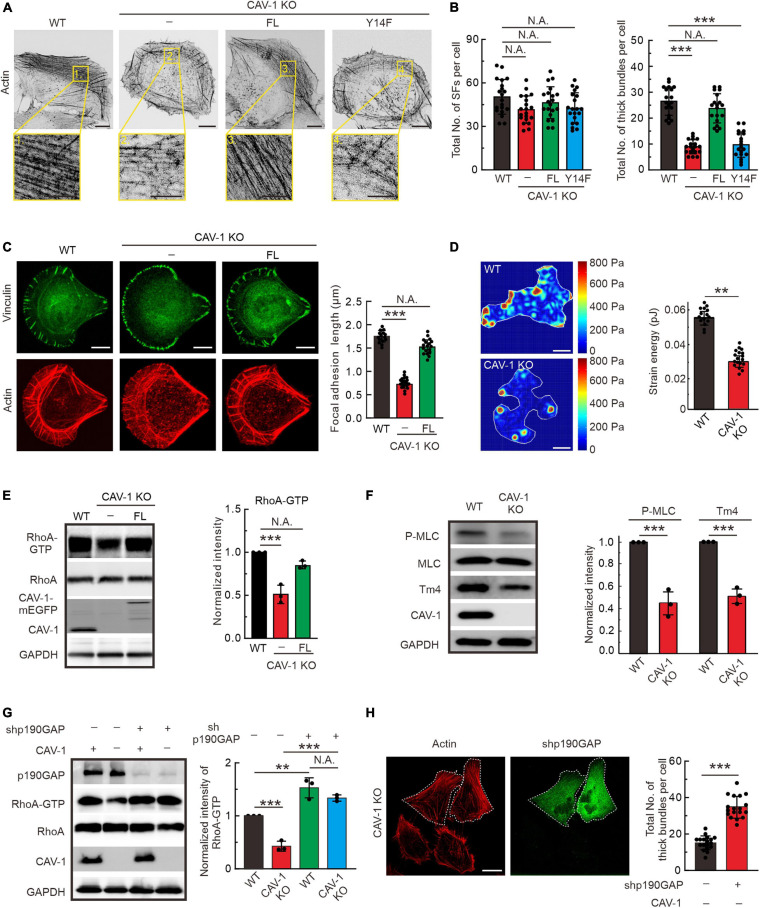
RhoA-Myosin II regulated assembly of contractile stress fibers is inhibited in CAV-1 KO cells. **(A)** Representative images of actin filaments visualized by phalloidin in WT, CAV-1 knockout (CAV-1 KO), full-length CAV-1(FL), and phospho-deficient CAV-1(Y14F) expressing cells. Magnified regions represent the contractile stress fiber. Bars, 10 μm (in cell images) and 5 μm (in the magnified box). **(B)** The average numbers of total and thick filaments were calculated for cells depicted in panel A. *n* = 20 cells from each group. **(C)** Representative images of actin filaments and focal adhesions visualized by phalloidin and vinculin antibody staining, respectively, in WT, CAV-1 KO cells, and CAV-1(FL)-mEGFP expressing cells cultured on crossbow shaped fibronectin coated micropatterns. Bars, 10 μm. Quantifications of focal adhesion lengths of each group are shown on the left. *n* = 15/17/18 from WT/CAV-1 KO/CAV-1(FL)-rescue cells. **(D)** Representative force maps of WT and CAV-1 KO U2OS cells grown on 25 kPa polyacrylamide dishes with fluorescent nanobeads. Bars, 10 μm. Quantification of contractile strain energy in WT (*n* = 15) and CAV-1 KO (*n* = 17) cells are shown on the right. **(E)** Pull-down assays were performed for WT, CAV-1 KO, and CAV-1(FL)-rescue cells. Proteins bound to GST-Rhotekin binding domain were analyzed by western blots and further quantified (compared to total RhoA) based on the band’s intensity. *n* = 3. **(F)** Western blot analysis and quantifications of phosphorylated MLC (Thr18/Ser19) (compared to total MLC) and Tpm4.2 in WT and CAV-1 KO cell lysates. *n* = 3. **(G)** WT and CAV-1 KO cells were transfected with Control shRNA and p190RhoGAP shRNA for 72 h, respectively. Cell lysis from each group were analyzed by western blots and further quantified based on the band’s intensity. *n* = 3. In panel **(E–G)**, the obtained intensity value from WT cells was set to 1. **(H)** CAV-1 knockout cells transfected with p190RhoGAP shRNA were stained by Alexa 568 phalloidin. The p190RhoGAP knockdown cells are marked by dotted lines. The average numbers of thick filaments were calculated on the right. *n* = 18 cells from each group. Data in panel **(B–H)** are presented as mean ± SD. In **(B,C,G)**, ***P < 0.001; **P < 0.01; N.A., not significant (one-way ANOVA). In **(D–F,H)**, ***P < 0.001; **P < 0.01; N.A., not significant (unpaired *t*-test). All the data are from three independent experiments.

To explore the cellular function of CAV-1, we generated CAV-1 knockout (KO) U2OS cells by CRISPR/Cas9 approach with two different target sites, and one of the CAV-1 KO cell lines was chosen for further analysis (Supplementary Figure 3A), and the rest cells were used for verification in some of the experiments (data not shown). Phalloidin staining was shown that wild-type cells contained prominent stress fibers, whereas both CAV-1 KO and RNA silencing induced CAV-1 knockdown (CAV-1 KD) cells contained a disorganized meshwork of actin filaments, characterized by thinner contractile stress fibers (Figure 3A and Supplementary Figures 3B,C). The quantification by using Ridge Detection plugin in ImageJ (Kumari et al., 2020; Zhao et al., 2020) confirmed that there was significant decrease in levels of thick actin filament bundles in CAV-1 KO/KD cells, whereas the total amount of filaments remained comparable (Figure 3B and Supplementary Figure 3C). To allow more precise analysis, cells were plated on crossbow shaped fibronectin micropatterns, where they obtain nearly identical shapes and display characteristic organization of stress-fiber network (Jiu et al., 2015). The contractile actomyosin bundles were typically thinner in CAV-1 KO cells compared to wild-type cells on micropatterns (Figure 3C). Moreover, CAV-1 KO cells displayed wider lamella which is a phenotype associated with defects in the assembly and contractility of stress fibers (Burnette et al., 2014; Jiu et al., 2015). Corresponding with the reduced amounts of thick stress fibers, the average sizes of vinculin-positive focal adhesions and phosphorylation level of focal adhesion kinase (FAK) were decreased in CAV-1 KO cells (Figure 3C and Supplementary Figure 2D). Importantly, exogenously expression of the full-length CAV-1-mEGFP completely restore the stress fiber and focal adhesion phenotypes, but not the phospho-deficient CAV-1(Y14F)-mEGFP (Figures 3A–C), indicating that Tyr14 is a critical site for CAV-1 and expression this single point mutation of CAV-1 recapitulates the CAV-1 KO phenotype of compromised stress fiber assemblies. Furthermore, CAV-1 deficient cells exhibited weaker contractile forces and an unbalanced tension distribution to the substrate (Figure 3D), which is the consequences from actomyosin defects.

Small GTPase RhoA regulates the phosphorylation of myosin light chain (MLC) to promote stress fiber contractility and assembly (Guilluy et al., 2011a, b; Lessey et al., 2012). By using pull-down assay and RhoA-GTP biosensor GFP-AHPH (Piekny and Glotzer, 2008; Rong et al., 2021), we revealed that the active level of GTP bound RhoA significantly decreased in CAV-1 KO cells, exogenously expression of CAV-1-mEGFP restore the active level of RhoA (Figure 3E and Supplementary Figure 3E). To confirm these observations, we tested the consequences downstream of RhoA. Phosphorylation of MLC and the level of tropomyosin (isoform Tpm4.2) which required for myosin II filament formation and integrity (Umemoto et al., 1989; Burgess et al., 2007; Geeves et al., 2015), also displayed significant reduction in CAV-1 KO cells (Figure 3F and Supplementary Figures 3F,G). p190RhoGAP is a major upstream negative regulator of Rho GTPases (Jaffe and Hall, 2005), knockdown of which restores the decreased RhoA-GTP level and the weaker stress fibers in CAV-1 depletion cells (Figures 3G,H). Collectively, these results reveal that CAV-1 is critical for the contractile stress fibers formation.

### Phospho-Deficient and Depletion of CAV-1 Leads to AMPK Activation Followed by Rac1-Dependent PAK1 and Cofilin Phosphorylation, and Subsequent Lamellipodia Formation

**FIGURE 4:**
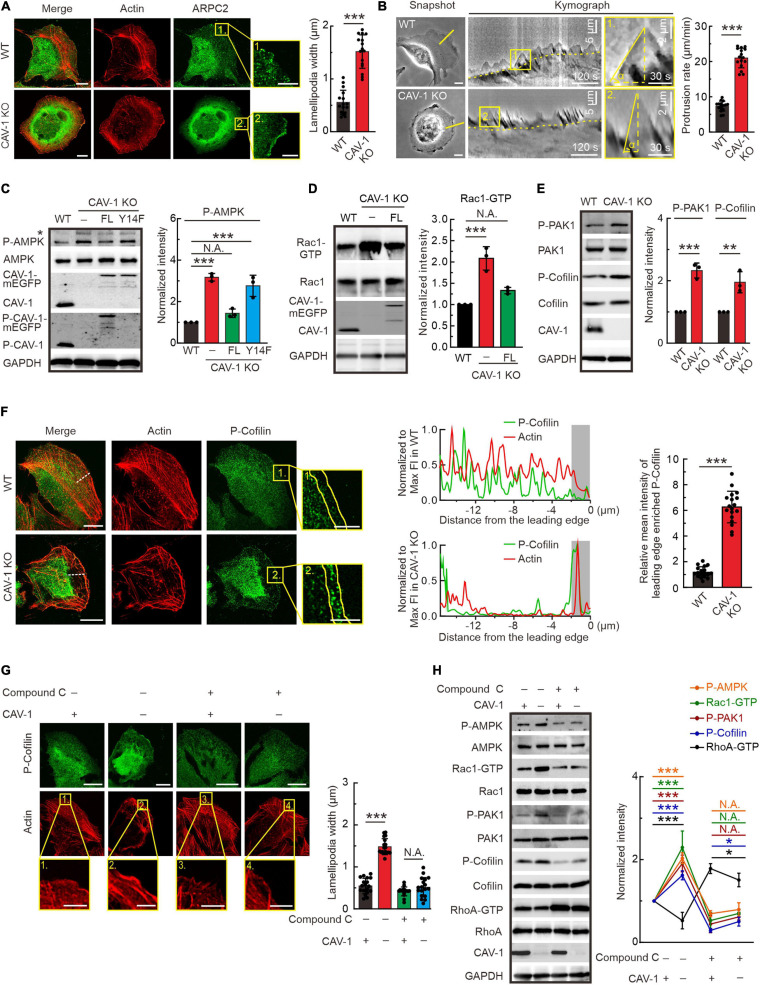
Actin protrusive network is upregulated by AMPK-Rac1-PAK1-Cofilin signaling cascade in cells lacking CAV-1. **(A)** Immunofluorescence microscopy analysis demonstrating that more pronounced endogenous ARPC2 colocalizes with F-actin (visualized by Alexa 568 phalloidin) on the lamellipodia protrusions in CAV-1 deficient cells. Magnified regions of cell edges on the right show the distribution of ARPC2 in WT and CAV-1 KO cells. Bars, 10 μm (in cell images) and 5 μm (in the magnified box). Quantification of width of lamellipodial protrusions are shown on the right. *n* = 16 regions from 16 cells for each group. **(B)** Representative images of membrane ruffling by time-lapse microscope. 1-pixel-wide areas were cut out to generate a 300 frame 2 s interval kymograph. Yellow dashed lines indicate the track of cell movement. An enlarged region is displayed on the right, vertical dashed lines show the membrane protrusion distance, while horizontal dashed lines mark the duration of protrusion. Bars, 10 μm (in cell images) and 2 μm (in the magnified box). Quantification of protrusion rate are shown on the right. *n* = 16 regions from 16 cells for each group. **(C)** P-AMPK (Thr172) and total AMPK were detected from the lysates of each group by western blotting. Please note that CAV-1(Y14F)-mEGFP can’t be detected by using phospho-CAV-1(Tyr14) antibody. Asterisk denotes the non-specific band. Quantification of P-AMPK (Thr172) levels (compared to total AMPK) from each group was shown on the right panel. *n* = 3. **(D)** Pull-down assays were performed for WT, CAV-1 KO, and CAV-1 KO; CAV-1-mEGFP re-expressed cells. Proteins bound to GST-PAK binding domain were analyzed by western blots and further quantified (compared to total Rac1) based on the band’s intensity. *n* = 3. **(E)** Western blot analysis and quantification (compared to total PAK1 and Cofilin) of the levels of phosphorylated PAK1 (Thr423) and Cofilin (Ser3) in WT and CAV-1 KO cell lysates. *n* = 3. **(F)** Immunostaining and quantification of endogenous P-Cofilin (Ser3) and F-actin distribution in WT and CAV-1 KO cells. A 16 μm length line was used to generate a line profile to illustrate the co-localization of P-Cofilin (Ser3) and F-actin. The lamellipodia region was enlarged on the right, and 2 μm width region was chosen to analyze the mean intensity of P-Cofilin (Ser3) on the leading edge. Bars, 10 μm (in cell images) and 5 μm (in the magnified box). *n* = 18 regions from 18 cells for each group. **(G)** Immunostaining and quantification of endogenous P-cofilin (Ser3) distribution upon compound C treatment in WT and CAV-1 KO cells. Magnified regions represent the lamellipodia region. Bars, 10 μm (in cell images) and 5 μm (in the magnified box). *n* = 18 regions from 18 cells for each group. **(H)** Western blot analysis and quantification of the phosphorylated levels of AMPK (Thr172), PAK1 (Thr423), Cofilin (Ser3) and activity of Rac1 and RhoA upon compound C treatment. *n* = 3. In panel **(C–E,H)**, the obtained intensity value from wild-type cells was set to 1. All the data are presented as mean ± SD. In **(C,G,H)**, ***P < 0.001; *P < 0.05; N.A., not significant (one-way ANOVA). In **(D–F)**, ***P < 0.001; **P < 0.01 (unpaired *t*-test). All the data are from three independent experiments.

It is important to note that in addition to disorganized stress fiber network, both CAV-1 KO and phospho-deficient CAV-1(Y14F) cells appear more pronounced lamellipodia in the cell edge visualized by actin and ARPC2 staining (Figures 3A, 4A). The actin distribution was further checked by interference reflection microscopy (IRM), which is applied to visualize molecules near the cell-substrate interactions (Verschueren, 1985). Consistent with aforementioned phalloidin staining, strong stress fibers were hardly observed in CAV-1 KO cells, while more prominent lamellipodia were localized around the leading edge in response to CAV-1 depletion (Supplementary Figure 4A). To test whether strong lamellipodia has consequences in membrane ruffling, we performed time-lapse analysis focusing on the cell edges and revealed that ruffling is much faster and broader, yet concomitantly not able to push membrane forward in CAV-1 KO cells, whereas the protrusive ruffling activity in wild-type cells display less frequency and occurs only in the direction of movement (Figure 4B and Supplementary Video 3).

AMP-activated protein kinase (AMPK) is a phylogenetically conserved intracellular energy sensor that has been shown to regulate small GTPases (Salani et al., 2012; Luo et al., 2018). By western blots, we noticed that the phosphorylation level of AMPK was significantly increased in CAV-1 KO cells, which could be rescued by full-length but not Y14F phospho-deficient CAV-1, furthering indicating the essential role of site Tyr14 of CAV-1 and confirming the actin associated phenocopy of CAV-1 KO with CAV-1 (Y14F) mutation (Figure 4C). In addition to RhoA, we also measured the GTP bound active levels of Rac1 and Cdc42, and found that CAV-1 depletion led to more active Rac1 and Cdc42 while their total protein levels remained unaffected (Figure 4D and Supplementary Figure 4B). It has been already known that Rac1 induce the formation of lamellipodia and surface protrusions generated by actin-remodeling reactions (Ridley et al., 1992, 2003). In this regard, the thicker lamellipodia and active membrane ruffling observed in CAV-1 KO cells fit well as a consequence of the active AMPK and Rac1.

Furthermore, we evaluated and found an increase in the basal activation of lamellipodia related PAK1 and Cofilin (Figure 4E). Importantly, the phosphorylated AMPK, PAK1 and Cofilin in CAV-1 KO cells prefer to localize more to the cell edges where lamellipodia form (Figure 4F and Supplementary Figures 4C–F). By treating with AMPK inhibitor compound C (Dasgupta and Seibel, 2018), both the distribution of phosphorylated Cofilin and lamellipodia width were restored to wild-type level in CAV-1 KO cells (Figure 4G), supporting the hypothesis that changes of actin protrusive network in the absence of CAV-1 is regulated by AMPK-Rac1-PAK1-Cofilin signaling cascade.

To test whether AMPK is the original cause for the actin network rearrangement in CAV-1 depletion cells, we blocked AMPK activation and revealed the clear recovery of increased Rac1-GTP and decreased RhoA-GTP, as well as elevated levels of phosphorylated PAK1 and Cofilin in CAV-1 KO background (Figure 4H). Taken together, we identified that sensitize AMPK mediated activities of GTPases in CAV-1 depletion cells play essential roles for actin cytoskeleton remolding.

### Cell Morphology and Directional Migration Are Impaired by Depletion of CAV-1

**FIGURE 5:**
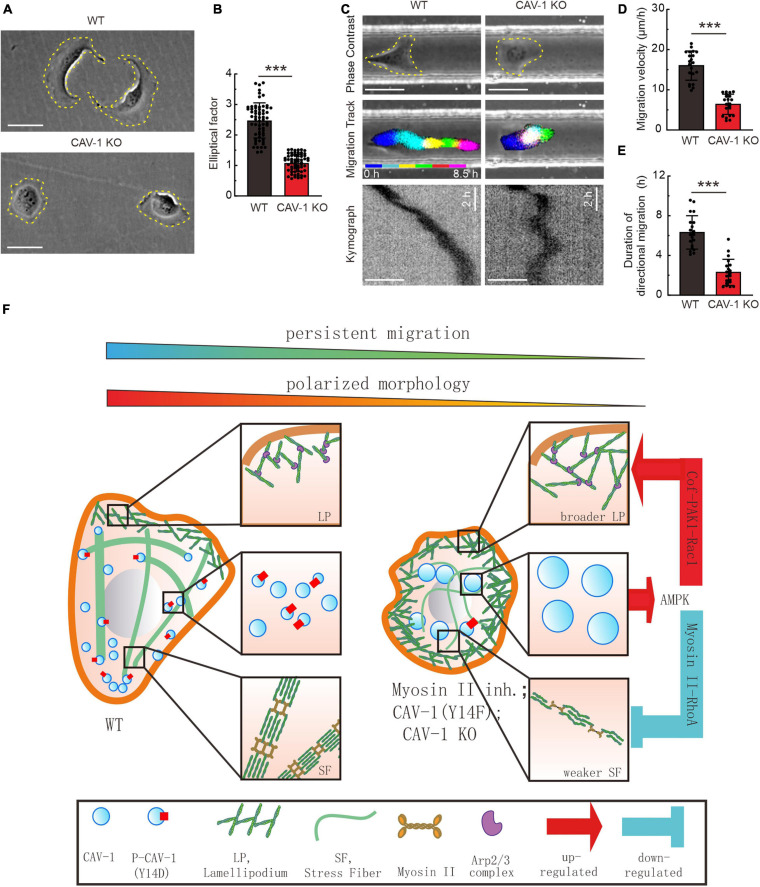
CAV-1 is required for the cell polarized morphology establishment and directional cell migration. **(A)** Phase contrast imaging reveals the morphology of WT and CAV-1 KO cells. Bars, 50 μm. **(B)** The elliptical factor (length/breadth) were calculated for WT (*n* = 63) and CAV-1 KO (*n* = 63) cells. **(C)** Representative images form time-lapse videos of WT and CAV-1 KO cells migrating in 50 μm width and 50 μm height channels with fibronectin-coated surface. Color lines indicate the trajectory of each single cell nucleus stained by Hoechst and the kymograph are shown in the lower panel. Bars, 50 μm. **(D,E)** Migration velocities **(D)** and directionalities **(E)** of WT and CAV-1 KO cells. Quantification is based on tracking of the displacement of nuclei. The data are presented as average velocity obtained from a 10 h cell tracking. *n* = 21 cells for each group in panel **(D)** and 20 cells in panel E. In **(B,D)**, ***P < 0.001 (Mann-Whitney test); In **(E)**, ***P < 0.001 (unpaired *t*-test). All the data are from three independent experiments. **(F)** A working model illustrates the negative feedback loop between actin assemblies and cytoplasmic CAV-1.

In order to explore whether the dysbiosis of actin assemblies in CAV-1 depletion cells affects cell shape and behavior, we recorded and measured cell morphology and directional cell migration. Most of wild-type U2OS cells exhibited a polarized morphology, with an elongated, polygonal shape. However, CAV-1 depletion cells adopted a rounded, non-polarized shape with lamellipodia encircling the entire cell (Figures 5A,B). Moreover, both the migration velocity and persistence were significantly reduced in CAV-1 KO cells quantified from cells on the custom-designed micro-channels and wound healing settings (Figures 5C–E, Supplementary Figures 5A,B, and Supplementary Video 4). Hepatocarcinoma Huh7 cells have no endogenous CAV-1 (Moreno-Caceres et al., 2017) (Supplementary Figures 5C,D) and stable exogenously expression of CAV-1-mEGFP led to increased would healing ability (Supplementary Figures 5E,F), further validating the role of CAV-1 in cell migration. Collectively, our results demonstrate that CAV-1 is required for the integrity and hemostasis of actin assemblies, and subsequent cell polarized morphology establishment and directional cell migration.

## Discussion

Despite accumulated information regarding the association between cortical actin filaments and membrane-located mechanosensitive caveolae with tension reservoir function (Sinha et al., 2011), here we propose a feedback-driven model which demonstrate that the interplay between cytoplasmic actin assemblies and phosphorylated CAV-1 coordinates cell migration (Figure 5F). Aside from plasma membrane bounded CAV-1, we observed that the cellular CAV-1 display actin associated motility by sliding along or/and undergoing retrograde flow with actin filaments. Adequate contractility of actin filaments is essential for the phosphorylation of CAV-1 on its critical site Tyr14, and the subsequent distribution and motility of the intracellular CAV-1 vesicles. Importantly, both CAV-1 depletion and CAV-1 phosphorylation on Tyr14 are able to affect the active levels of AMPK, RhoA/Rac1 GTPase, and further disrupt the homostasis of contractile and protrusive structures of cytoplasmic actin, which lead to significant changes in cell morphology and directional migration.

In view of live cell imaging findings that cytoplasmic CAV-1 vesicles not only move along actin filaments but also accompany with actomyosin transverse arcs for centripetal flow, it is tempting to speculate that these “tracking” and “anchoring” function of elongated actin filament bundles to CAV-1 vesicles perhaps requires molecular motor related contraction and consumes adenosine triphosphate (ATP) to implement these dynamic association. This speculation is coincided with our results that inhibiting the motor activity of myosin II significantly weakens the movement of the cytoplasmic CAV-1 vesicles (Figures 2C–E). Previous work by us reveals that vimentin intermediate filaments (IFs) function as physical barriers to restrain the intracellular trafficking of CAV-1 vesicles (Jiu, 2018; Shi et al., 2020). It is thus possible that the overall integrity and motility pattern of the cytoplasmic CAV-1 are comprehensively regulated by both actin and IFs cytoskeletal networks. Moreover, trafficking of CAV-1 from plasma membrane to perinuclear area has been reported by far dependent on microtubules (Conrad et al., 1995; Mundy et al., 2002; Echarri et al., 2012; Echarri and Del Pozo, 2015). Therefore, our finding which associates contractile actin filaments with dynamics of cellular CAV-1 vesicles appears as a novel and complementary observation and will contribute to characterize the integrated cycling of caveolae. In addition, early studies have shown that the motility of CAV-1 is bound to depolymerized actin fibers by cytochalasin D application (Mundy et al., 2002; Thomsen et al., 2002; Stoeber et al., 2012). Combined with our results, we argue that the cytoplasmic distribution and dynamics of caveolae are mostly dependent on the contractile and filamentous status of actin, which representing the abundant and stiff of actin structures.

We assume that CAV-1 depleted U2OS cells which used in this study are considered to be deprivation of all three caveolin genes, because (1) CAV-2 has been shown to be degraded in the absence of CAV-1 through the proteasomal pathway (Razani et al., 2002a), and (2) CAV-3 is a muscle specific caveolin which showed no endogenous expression in U2OS cells (Galbiati et al., 2001; Razani et al., 2002b). In line with our finding, activation of AMPK by CAV-1 depletion has been also detected in other human colon tumor cells (Ha et al., 2012), indicating the activity control of CAV-1 to AMPK is a broad regulation in cancer cells. Moreover, depletion of CAV1 led to AMPK activation followed by a p53-dependent G1 cell-cycle arrest and autophagy, suggesting that elevated CAV1 may contribute to the ATP generation (Ha et al., 2012).

This is already a common view that there is mutual inhibition between the lamellipodia formation in cell frontness and the contractile stress fiber formation in cell backness (Li et al., 2003; Meili and Firtel, 2003; Xu et al., 2003) during persistent migration. Thus, we propose that CAV-1 depletion disequilibrates the balance between RhoA-mediated actomyosin bundles and Rac1-mediated lamellipodia formation, which potentiates the interpretation of the cell migration defects. Furthermore, here we identify a previously unexpected role for CAV-1 phosphorylation on site Tyr14 in inhibition of AMPK, and also disrupt the GTPase homeostasis and eventually cell migration.

Other plasma membrane domains, such as clathrin-coated pits are functionally linked to actin-regulatory factors (Girao et al., 2008; Grassart et al., 2014; Tweten et al., 2017), but an alignment with actin filaments is not apparent in those invaginations, suggesting that the association of caveolae with actin filaments fulfills a particular function, and that differentiates them from clathrin-coated pits. It will be interesting to explore in the future why caveolae associate with certain actin filament structures but not with others, when and how this association is stimulated and regulate various cell behavior.

## Materials and Methods

### Cell Culture and Transfections

Human osteosarcoma (U2OS) cells and human hepatocarcinoma (Huh7) cells were maintained in high glucose (4.5 g/L) Dulbecco’s modified Eagle’s medium (DMEM) (#06-1055-57-1A, Biological Industries, Kibbutz Beit-Haemek, Israel) supplemented with 10% fetal bovine serum (FBS, #10270-106, Gibco, Waltham, MA, United States), 100 U/ml Penicillin, 100 μg/ml Streptomycin, and 4 mM L-Glutamine (later referred as complete DMEM) at 37°C in humidified atmosphere with 5% CO_2_. Transient transfections were performed with Fugene HD (Promega, Madison, WI, United States) according to manufacturer’s instructions using a Fugene HD: DNA ratio of 3.5:1 and 24 h incubation prior assay. siRNA experiments were performed with Lipofectamine RNAiMAX (Invitrogen, Carlsbad, CA, United States) using 40 nM On-target plus human siRNA of CAV-1 (target sequence 5′ CCCUAAACACCUCAACGAU 3′), and ARPC2 (target sequence 5′ CCAUGUAUGUUGAGUCUAA 3′) (Dharmacon, Lafayette, CO, United States) for 72 h, respectively. AllStars Neg. Control siRNA (Qiagen, Hilden, Germany) was used as a control siRNA.

### CAV-1 CRISPR Knockout Cell Line Generation

CAV-1 knockout U2OS cell line was generated based on pSpCas9 (BB)-2A-GFP vector (a gift from Feng Zhang, #48138, Addgene, Cambridge, MA, United States). Briefly, two guide sequence targeting of human CAV-1 were selected based on CRISPR Design Tool^1^ with the primer: 5′ AGTGTACGACGCGCACACCA 3′ and 5′ TGGGGGCAAATACGTAGACT 3′ for CAV-1-knockout. Transfected cells were detached at 24 h post-transfection and sorted with FACS Aria II (BD Biosciences, Bedford, MA, United States), using low intensity GFP-expression pass gating, as single cell onto 96-well plate supplemented DMEM containing 20% FBS and 10 mM HEPES. CRISPR clones were cultivated for 2 weeks prior selecting clones with no discernible CAV-1 protein expression using western blotting.

### Immunofluorescence Microscopy

Immunofluorescence (IF) experiments were performed as previously described (Jiu et al., 2019). Briefly, cells were fixed with 4% PFA in PBS for 15 min at room temperature (RT), washed three times with PBS, and permeabilized with 0.1% Triton X-100 in PBS for 5 min. Cells were then blocked in PBS supplemented with 5% BSA. Both primary and secondary antibodies were applied onto cells and incubated at RT for 2 h. Alexa-conjugated phalloidin was added together with secondary antibody solutions onto cells. Alexa 647-wheat germ agglutinin (WGA) (#W32466, Thermo Fisher Scientific) was used to visualize the plasma membrane. All IF data were obtained with Olympus SpinSR10 Ixplore spinning disk confocal microscope with UplanApo 100×/1.5 Oil objective (Olympus Corporation, Tokyo, Japan). The pixel size was optimized properly to achieve the maximum resolution which was calculated to be 65 nm. For detection and measure of the cytoplasmic CAV-1 tagged vesicles, the “Spots” tool of Imaris 9.2 (Bitplane, Zurich, Switzerland) was used with the configuration defined as 2 μm for estimated XY diameter. The numbers and sizes of spots were calculated subsequently. For detection and measure of the lamellipodia width, a plot profile perpendicular to the plasma membrane was made by using imageJ, and the peak zone was defined as the width.

### Live Cell Imaging

For live cell imaging, 35 mm glass-bottomed dishes (MatTek Corporation, Ashland, MA, United States) were coated with 10 μg/ml fibronectin (#F2006, Sigma Corp., St. Louis, MO, United States) in PBS for at least 3 h at 37°C, washed with PBS twice and immersed in complete DMEM medium without phenol red (#01-053-1A, Biological Industries, Kibbutz Beit-Haemek, Israel) before seeding of cells. The time-lapse images of cells with transient transfection of CAV-1-mEGFP and mCherry-actin were acquired with Olympus cellSens Dimension system, consisting of an Olympus SpinSR10 Ixplore spinning disk confocal and a Yokogawa CSU-W1 confocal scanner. Appropriate filters, heated sample environment (+37°C), controlled 5% CO_2_ and UplanApo 100×/1.5 Oil objective (Olympus Corporation, Tokyo, Japan) was used. The recording was set as every 1 s for 200 s and one focal plane was recorded for all live cell videos. For tracking and speed measurement of CAV-1 vesicles, the Imaris 9.2 (Bitplane, Zurich, Switzerland) “Track” module with globular-objects over time was used as in previous study (Jiu, 2018). Two micrometers estimated XY diameter, 5 μm max distance and 3 max gap size were set for analyzing.

### Interference Reflection Microscopy (IRM)

In order to image cells using IRM, we added a 50/50 beam splitter (which reflects 50% and transmits 50% of the chosen wavelength) in an empty filter cube of the Olympus IX73 inverted widefield fluorescence microscopy (Olympus Corporation, Tokyo, Japan). The 50/50 beam splitter partially reflects the 488 nm light to the sample, and then the light reflected from the sample is collected by the camera. The light reflected from the glass/medium interface (I1) and the light reflected from the medium/cell plasma membrane interface (I2) can interference, the optical path difference between I1 and I2 will result in a constructive bright signal or a destructive dark signal (Verschueren, 1985; Mahamdeh and Howard, 2019).

### Western Blotting (WB)

All cell lysates were prepared by washing the cells once with PBS and scraping them into RIPA lysis buffer (50 mM Tris pH 7.4, 150 mM NaCl, 1% Triton X-100, 1% sodium deoxycholate and 0.1% SDS) supplemented with 1 mM PMSF, 10 mM DTT, 40 μg/ml DNase I and 1 μg/ml of leupeptin, pepstatin, and aprotinin. All preparations were conducted at 4°C. Protein concentrations were determined with BCA Protein Assay kit (#23227, Thermo Fisher Scientific, Waltham, MA, United States) and equal amounts of the total cell lysates were mixed with Laemmli Sample Buffer (LSB), boiled, and ran on 12.5% SDS-PAGE gels. Proteins were transferred to nitrocellulose membrane with Trans-Blot Turbo transfer system (Bio-Rad, Hercules, CA, United States) using Mini TGX gel transfer protocol. Membrane was blocked in 5% BSA for 1 h at RT. Primary and secondary antibodies were diluted into fresh blocking buffer for overnight at 4°C and 1 h at RT, respectively. Proteins were detected from the membranes with SuperSigna West Femto Maximum Sensitivity Substrate (Thermo Fisher Scientific, Waltham, MA, United States).

### Plasmids

mCherry-actin was a kind gift from Pekka Lappalainen (University of Helsinki, Finland). CAV-1-mCherry (#27705), CAV-1-mEGFP (#27704) and GFP-AHPH (#Cat68026) were from Addgene (Watertown, MA, United States). All plasmids were sequenced for verification. CAV-1(Y14D)-mEGFP and CAV-1 (Y14F)-mEGFP was constructed by the overlap extension method, respectively (Horton et al., 1989). To knockdown p190RhoGAP in U2OS cells, human p190RhoGAP (GenBank NM_004491.4) targeting sequence (5′ CCGGCGGTTGGTTCATGGGTACATTCTCGAGAATGTAC CCATGAACCAACCGTTTTTT 3′) and control, non-targeting sequence (5′ CCGGGGTTCTCCGAACGTGTCACGTCTCGA GACGTGACACGTTCGGAGAACCTTTTTG 3′) were cloned into pLKO.1-TRC-copGFP-T2A-Puro vector, respectively.

### Drug Treatment

The following drugs were used at a defined dose and time: blebbistatin (#b0560; Sigma, St. Louis, MO, United States) with 10 μM for 30 min, CK666 (#sml0006, Sigma, St. Louis, MO, United States) with 40 μM for 1 h, Latrunculin B (LatB, #sc-203318, Santa Cruz, Dallas, TX, United States) with 0.5 μM for 0.5 h and Compound C (#S7306, Selleck Chemicals, Houston, TX, United States) with 5 μM for 24 h.

### Antibodies

The following antibodies were used in this study: CAV-1 (D46G3) rabbit antibody (1:1,000 dilution for WB, 1:200 for IF; #3267, Cell Signaling, Beverly, MO, United States); Phospho-CAV-1 (Tyr14) rabbit antibody (dilution 1:1,000 for WB; #3251, Cell signaling); AMPK rabbit antibody (dilution 1:500 for WB; #SAB4502329, Sigma, St. Louis, MO, United States); P-AMPK (Thr172) rabbit antibody (dilution 1:500 for WB, 1:100 for IF; #2531S, Cell Signaling); Cofilin (E-8) mouse antibody (dilution 1:1,000 for WB; #sc-376476, Santa Cruz, Dallas, TX, United States); Phospho-Cofilin (Ser3) rabbit antibody (dilution 1:1,000 for WB, 1:200 for IF; #3313, Cell signaling); p190RhoGAP rabbit antibody (dilution 1:2,000 for WB; #26789, Proteintech, Rosemont, IL, United States); FAK rabbit antibody (dilution 1:1,000 for WB; #3285, Cell Signaling); Phospho-FAK (Tyr397) rabbit antibody (dilution 1:1,000 for WB; #3283, Cell Signaling); PAK1 rabbit antibody (dilution 1:1,000; #2602, Cell Signaling); Phospho-PAK1 (Thr423)/PAK2 (Thr402) rabbit antibody (dilution 1:1,000 for WB, 1:200 for IF; #2601, Cell Signaling); Tpm4.2 (LC24) mouse antibody (dilution 1:500 for WB and IF; a kind gift from Peter W. Gunning, UNSW Australia); Phospho-myosin light chain 2 (Thr18/Ser19) rabbit antibody (dilution 1:500 for WB, 1:200 for IF; #3674, Cell Signaling); Myosin light chain mouse antibody (dilution 1:1,000 for WB; #M4401, Sigma); Vinculin mouse antibody (dilution 1:100 for IF; #V9131, Sigma); ARPC2 rabbit antibody (dilution 1:1,000 for WB and IF; #15058, Proteintech, Rosemont, IL, United States); Myosin-18B rabbit antibody (dilution 1:500 for WB; #HPA000953, Sigma); Rab8 rabbit antibody (dilution 1:100 for IF; #R5530, Sigma); and GAPDH mouse polyclonal antibody (dilution 1:1,000 for WB; #G8795, Sigma).

### FRAP

Cells were transfected with CAV-1-mEGFP and incubated for 24 h. Confocal images were acquired with a 3I Marianas imaging system (3I Intelligent Imaging Innovations, Denver, CO, United States), consisting of an inverted spinning disk confocal microscope Zeiss Axio Observer Z1 (Zeiss, Oberkochen, Germany), a Yokogawa CSU-X1 M1 confocal scanner and 63 × /1.2 WC-Apochromat Corr WD = 0.28 M27 objective (Zeiss). Heated sample environment (+37°C) and 5% CO_2_ control were used. SlideBook 6.0 software (3I Intelligent Imaging Innovations) was used for the image acquirement. Five pre-bleach images were acquired followed by bleaching scans with 100% intensity laser lines over the region of interest. Recovery of fluorescence was monitored 50 times every 200 ms and 300 times every 1 s. The intensity of the bleached area was normalized to a neighboring non-bleached area. Mean scatter plots were calculated from different FRAP experiments and the means and standard deviations were calculated.

### Filament Analysis

The total number of stress fibers and the number of thick actin filament bundles in U2OS cells were quantified with ridge detection plugin (v1.4.0) from Fiji ImageJ (1.53c, Wayne Rasband, National Institutes of Health NIH). The parameters used for detecting the total number of stress fibers are: line width 20.0, high contrast 230, low contrast 100, sigma 6.57, low threshold 0.0, and upper threshold 0.34. The parameters used for quantifying the thick bundles are: line width 29.0, high contrast 230, low contrast 87, sigma 8.87, low threshold 0.0, and upper threshold 0.17. The dorsal stress fibers in cells on petri dishes and the ventral stress fibers in cells on micropattern chips are manually outlined, and subsequently measured the lengths for quantification.

### BioID Screen

BirA-Tpm3.1 was transfected into U2OS cells, which induced biotinylation of transverse arcs and ventral stress fibers (Supplementary Figure 1A). Backbone vector pmycBioID-C1 was transfected as control. Cells were grown for 24 h in complete DMEM and another 24 h in the presence of 50 μM biotin. Single-step affinity purifications of the biotinylated proteins, liquid chromatography mass spectrometry sample preparation, and mass spectrometry were performed as in Jiu et al. (2015). To obtain a list of high-confidence protein interactions for Tpm3.1 the data was filtered against our in-house BioID contaminant database.

### Real-Time Quantitative PCR

Total mRNA was extracted with GeneJET RNA purification kit (#K0731, Thermo Fisher Scientific, Madison, WI, United States) and single stranded cDNA was synthetized (#K1671, Thermo Fisher Scientific) from 500 ng of extracted mRNA. The following primers were used: forward CAV-1 5′ AACCTCCTCACAGTTTTCATCC 3′, reverse CAV-1 5′ CTTGTTGTTGGGCTTGTAGATG 3′, forward GAPDH 5′ GAAGGTGAAGGTCGGAGTC 3′, reverse GAPDH 5′ GAAGATGGTGATGGGATTTC 3′. Quantitative PCR reactions were carried out with Maxima SYBR Green/ROX (#K0221, Thermo Fisher Scientific) in Bio-Rad CFX96 (Bio-Rad). Changes in expression were calculated with 2^–ΔΔ*Ct*^ method, and normalized to GAPDH and WT expression levels, respectively.

### Cell Migration Assay

The microfluidic device is made of PDMS (RTV615, NY, United States) by soft lithography from a patterned SU-8 silicon wafer. Glass coverslips were plasma bounded to PDMS layer. Each device consists of 10 channels of 50 μm in wide and height, 3 mm in length. In migration assay, all channels were coated with 10 μg/ml fibronectin for 1 h at 37°C. Cells were incubated with Hoechst 33342 for 10 min and subsequently washed twice with PBS and replaced with complete DMEM. Cells were collected from culture dishes using trypsin-EDTA, and resuspended in complete DMEM to a concentration of 2 × 10^7^ cells/ml. Twenty microliters of cell suspension was added to the device inlet. Cells were allowed to adhere and spread overnight. All wells of the device were then filled with 120 ml of complete DMEM. Devices were incubated at 37°C and 5% CO_2_ before imaging by Olympus IX73 inverted microscopy with the UplanFL 10 × /0.3 objective (Olympus, Tokyo, Japan). Average migration velocity and directional migration duration were quantified by tracking the nucleus movement in between 5 min imaging cycles for 10 h. Only cells that did not collide with one another were selected for measurements.

### Wound Healing Assay

Cells were seeded in a 6-well cell culture plate with a cell density of 25,000/cm^2^ and cultivated at 37°C in 5% CO_2_ overnight. Subsequently, the cell monolayers were scratched with a sterile 0.2 mL pipette tip to create linear wounds and washed with PBS to remove detached cells. Cells were incubated in a serum-free DMEM medium to eliminate cell proliferation and then observed by Olympus IX73 inverted microscopy with the UplanFL 10×/0.3 objective (Olympus, Tokyo, Japan). The migration rates were measured using ImageJ.

### Small GTPase Activity Assay

Active RhoA, Rac1 and Cdc42 Detection Kit (#8820, #8815 and #8819, Cell Signaling, Beverly, MO, United States) were used to measure the activity of these small GTPases. Briefly, cell lysates were prepared by washing cells once with ice-cold PBS and scraping them into lysis buffer plus 1 mM PMSF. Next, transfer the cell lysate to the spin cup which contains 100 μl agarose beads and 400 μg GST-Rhotekin-RBD (for binding to RhoA-GTP) or GST-PAK1-PBD (for binding to Rac1/Cdc42-GTP). Incubate the reaction mixture at 4°C for 1 h with gentle rocking. Then wash the beads three times with washing buffer and incubated with reducing sample buffer for 2 min at RT. Centrifuge the tube at 6,000 g for 2 min and heat the eluted samples for 5 min at 100°C. Anti-Rho, anti-Rac1 and anti Cdc42 antibodies (#8789, #8631 and #8747, Cell Signaling) were used to test active RhoA, Rac1, and Cdc42 by western blot, respectively.

### Traction Force Microscopy

Traction force microscopy was used to measure the contractile forces that cells exerted upon their substrate as previously described (Jiu et al., 2017). Briefly, cells were cultured for 3–8 h on custom-made 35-mm dishes (Matrigen Life Technologies, CA, United States) with fibronectin-coated PAA gel with either 25 or 0.5 kPa stiffness. The diameter of 200 nm yellow-green fluorescent (505/515) microspheres was immobilized to the surface of the gel. Images of the cells and the fluorescent microspheres directly underneath the cells were acquired during the experiments and after cell detachment with trypsin. By comparing the reference image with the experimental image, we computed the cell-exerted displacement field. From the displacement fields, and manual traces of the cell contours, together with knowledge of substrate stiffness, we computed the traction force fields using the approach of constrained Fourier-transform traction cytometry. From the traction fields, we calculated the strain energy by equation

$$U=\frac{1}{2}\,\int \text{T}\,\left(r\right).\text{u}\,\left(r\right)\,d\,A$$

It was the total deformation energy produced by the cells through applying the traction on the surface of the substrate, which suggested an integrated measure of cell traction.

### Statistical Analysis

Statistical data analyses were performed with Excel (Microsoft, Redmond, WA, United States) and Graphpad Prism 8 (GraphPad Software, La Jolla, CA). Normality of the data was examined with the Shapiro-Wilk test and a quantile-quantile plot. For the data following normal distribution, Student’s two-sample unpaired *t*-test was used. If data did not follow normal distribution, Mann-Whitney *u*-test for two independent samples was conducted. One-way ANOVA followed by a Tukey’s *post-hoc* test was used to evaluate differences between three or more groups. For analyzing the CAV-1 movement rate, the mean speed of each vesicle within 200 s live cell video was measured by Imaris “Track” module and pooled together to calculate the average rate with color bars indicating the tracked mean speed ranging from 0 to 0.4 μm/s (Figure 2F).

## Data Availability Statement

All datasets generated for this study are included in the article/Supplementary Material, further inquiries can be directed to the corresponding author/s.

## Author Contributions

XS carried out the majority of the experiments and interpretation of the data. ZW performed part of the data analysis. XS and KS participated in designing of the study. YW and Y-JL fabricated the micro-channel for cell migration assay. YJ conceived the study and wrote the manuscript with contributions from all other authors. All authors contributed to the article and approved the submitted version.

## Conflict of Interest

The authors declare that the research was conducted in the absence of any commercial or financial relationships that could be construed as a potential conflict of interest.

## Footnotes

1 crispr.mit.edu

## Supplementary Material

Supplementary Figure 1 The expression and motility of CAV-1 vesicles in U2OS cells. **(A)** A schematic cartoon demonstrates that tropomyosin is a tightly actin associated components. Immunofluorescence staining of Biotin fused Tpm3.1 and streptavidin localized to actin filaments to verify the screen are shown in the lower panel. Bars, 10 μm. **(B)** Representative immunofluorescence images of overexpressing CAV-1-mCherry paired with endogenous CAV-1 and Rab8 antibodies, respectively. The yellow box in the merged images show magnified images. Bars, 10 μm (in cell images) and 5 μm (in the magnified box). **(C)** Localization of endogenous CAV-1 and actin filaments in U2OS cells detected by CAV-1 antibody and fluorescent phalloidin, respectively. WGA was used to label the plasma membrane. Magnified regions from the area indicated by white boxes demonstrate that cytoplasmic CAV-1 aligns with actin filaments. The orthogonal view shows the view of the enlarged region, where “XY” and “XZ” indicated different cross-sections. **(D)** Representative images of actin filaments and endogenous CAV-1. DAPI was used to mark the nucleus. “Middle” refers to the cytoplasmic field of the cell, and “Bottom” indicates the ventral plasma membrane area. Bars, 10 μm (in cell images), 5 μm (in magnified images and orthographic views).

Supplementary Figure 2 Actin assemblies regulate the size and dynamics of CAV-1 vesicles. **(A)** Immunofluorescence staining of endogenous F-actin and CAV-1 positive vesicles in U2OS cells treated with 0.2 μM LatB for 30 min. Magnified regions represent the cytoplasmic CAV-1. Bars, 10 μm (in cell images) and 5 μm (in the magnified box). **(B)** The length distribution of CAV-1 vesicles. The number of vesicles in each group of size is divided by the total CAV-1 number of the same cell. *n* = 6,432 vesicles from 12 WT cells, and 9,543 vesicles from 18 LatB treatment cells. **(C,D)** The transcriptional and translational level of CAV-1 is examined by RT-PCR **(C)** and western blot **(D)** in total cell lysates of WT, NMII inh. and Arp2/3 inh. cells. **(E)** Western blot analysis of endogenous Myosin-18B (Myo18B) level in total cell lysates of WT and Myo18B KO cells by CRISPR/Cas9 methods. **(F)** Western blot analysis of endogenous ARPC2 level in total cell lysates of WT and ARPC2 knockdown (ARPC2 siRNA) cells. **(G)** The length distribution of CAV-1 vesicles. The number of vesicles in each size group is divided by the total CAV-1 number of the same cell. *n* = 17,902 vesicles from 27 cells (WT), 15,434 vesicles from 33 cells (Myo18B KO), and 14,434 vesicles from 23 cells (ARPC2 siRNA). **(H)** Quantification of the movement rate of CAV-1 vesicles in the whole WT (*n* = 31), Myo18B KO (*n* = 28) cells, and ARPC2 siRNA (*n* = 28) cells. All the data are presented as mean ± SD. In **(E,G)**, ****P* < 0.001; N.A., not significant (one-way ANOVA). All the data are from three independent experiments.

Supplementary Figure 3 CAV-1 is critical for the contractile stress fiber formation by regulating RhoA-Myosin II. **(A)** Western blot analysis of endogenous CAV-1 levels in total cell lysates of WT and CAV-1 CRISPR/Cas9 knockout cells. **(B)** Western blot analysis of endogenous CAV-1 levels in total cell lysates upon RNA silencing. **(C)** Representative images of actin filaments visualized by phalloidin in WT and CAV-1 knockdown cells. Magnified regions represent the contractile stress fibers and protrusive lamellipodia. Bars, 10 μm (in cell images) and 5 μm (in the magnified box). Quantification of the average numbers of total and thick filaments, and lamellipodia width in 20 WT and 20 CAV-1 knockdown cells. **(D)** P-FAK (Tyr397) and total FAK were detected from the lysates of each group by western blotting. Quantification of P-FAK (Tyr397) levels (compared to total FAK) from each group was shown on the right panel. *n* = 3. **(E)** WT and CAV-1 KO cells transfected with active RhoA sensor GFP-AHGP were stained by Alexa 568 phalloidin. Bars, 10 μm. The average intensity of GFP-AHPH were calculated on the right. *n* = 20 for WT and CAV-1 KO cells. **(F)** WT and CAV-1 KO cells stained with phalloidin and P-MLC (Thr18/Ser19) antibody demonstrate decreased phosphorylation of P-MLC in cells lacking CAV-1. Bars, 10 μm (in cell images) and 5 μm (in the magnified box). The average intensity of P-MLC was calculated on the right. *n* = 19 for groups depicted on the left. **(G)** Representative images and quantification of Tm4 in WT and CAV-1 KO cells. *n* = 21 for each group. All the data are presented as mean ± SD. In **(C–E,G)**, ****P* < 0.001; ***P* < 0.01; N.A., not significant (unpaired *t*-test). In **(F)**, ****P* < 0.001 (Mann-Whitney test); All the data are from three independent experiments.

Supplementary Figure 4 Changes of actin protrusive network in the absence of CAV-1 is regulated by AMPK-Rac1-PAK1-Cofilin signaling cascade. **(A)** Representative images of actin filaments visualized by Interference Reflection Microscopy (IRM). Magnified regions represent the contractile stress fibers and protrusive lamellipodia. Bars, 10 μm (in cell images) and 5 μm (in the magnified box). **(B)** Pull-down assays were performed for WT and CAV-1 KO cells. Proteins bound to GST-PAK binding domain were analyzed by western blots and further quantified (compared to total Cdc42) based on the band’s intensity. *n* = 3. **(C,D)** Immunostaining **(C)** and quantification **(D)** of endogenous P-AMPK (Thr172) and F-actin distribution in WT and CAV-1 KO cells. A 16 μm length line was used to generate a line profile to illustrate the co-localization of P-AMPK (Thr172) and F-actin. The lamellipodia region was enlarged on the right, and 2 μm width region was chosen to analyze the mean intensity of P-AMPK (Thr172) on the leading edge. Bars, 10 μm (in cell images) and 5 μm (in the magnified box). *n* = 20 regions from each group. **(E,F)** Immunostaining **(E)** and quantification **(F)** of endogenous P-PAK1 (Thr423) and F-actin distribution in WT and CAV-1 KO cells. A 16 μm length line was used to generate a line profile to illustrate the co-localization of P-PAK1 (Thr423) and F-actin. The lamellipodia region was enlarged on the right, and 2 μm width region was chosen to analyze the mean intensity of P-PAK1 (Thr423) on the leading edge. Bars, 10 μm (in cell images) and 5 μm (in the magnified box). *n* = 20 regions from each group. All the data are presented as mean ± SD. In **(B,D,F)**, ****P* < 0.001 (unpaired *t*-test). All the data are from three independent experiments.

Supplementary Figure 5 Cell morphology and directional migration are impaired in response to CAV-1 deficiency. **(A)** Wound healing assay was performed in WT and CAV-1 KO cells. The representative images in different time point during wound healing were shown. Bars, 100 μm. **(B)** Quantitation of the averaged wound healing rate. **(C)** Western blot analysis of endogenous CAV-1 and stable expressed CAV-mEGFP in Huh7 cells. **(D)** Representative images of CAV-1-mEGFP in non-transfected WT and CAV-1-mEGFP stable expressed Huh7 cells. Bars, 10 μm. **(E,F)** Representative images **(E)** and measurement of the wound healing rate **(F)** of WT and CAV-1-mEGFP stable expressed Huh7 cells. Bars, 100 μm. All the data are presented as mean ± SD. ***P* < 0.01 (unpaired *t*-test). All the data are from three independent experiments.

Supplementary Video 1 Related to Figure 1C (Box 1). Time-lapse video of CAV-1-mEGFP sliding along actin filaments in U2OS Cells. White and yellow arrows indicate the starting and ending position of discrete CAV-1 tagged vesicles, respectively. The display rate is 3 frames per second. Bar, 5 μm.

Supplementary Video 2 Related to Figure 1C (Box 2). Time-lapse video of CAV-1-mEGFP associated with contractile actin transverse arcs and displayed retrograde flow toward the cell center in U2OS Cells. White and yellow arrows indicate the starting and ending position of discrete CAV-1 tagged vesicles, respectively. The display rate is 15 frames per second. Bar, 5 μm.

Supplementary Video 3 Related to Figure 4B. Live cell phase contrast imaging of WT (left) and CAV-1 KO (right) cells membrane ruffling for 10 min with the time interval of 2 s. The display rate is 15 frames per second. Bar, 10 μm.

Supplementary Video 4 Related to Figure 5C. Live cell phase contrast imaging of WT (upper) and CAV-1 KO (lower) cells migrating in 50 μm height and width channel for 10 h with the time interval of 5 min. Nucleus were stained by Hoechst. The display rate is 15 frames per second. Bar, 50 μm.
