# Supplementary figures and images for: Feedback-Driven Mechanisms Between Phosphorylated Caveolin-1 and Contractile Actin Assemblies Instruct Persistent Cell Migration
Source: Front Cell Dev Biol. 2021 Apr 12;9:665919. doi: 10.3389/fcell.2021.665919 (PMC8076160; doi:10.3389/fcell.2021.665919)

Figure S1

A

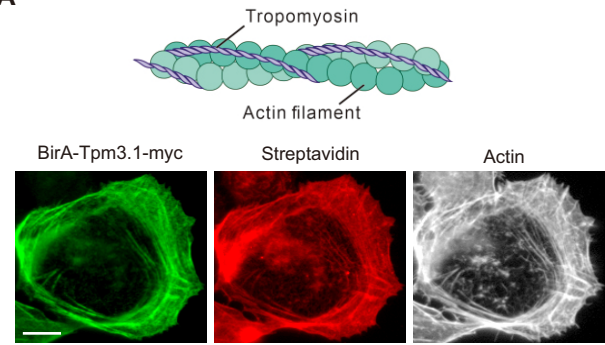

B

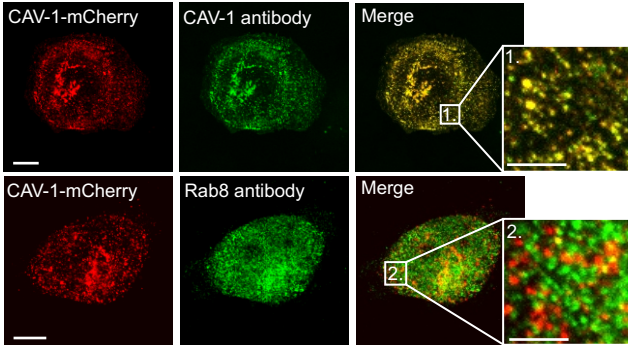

C

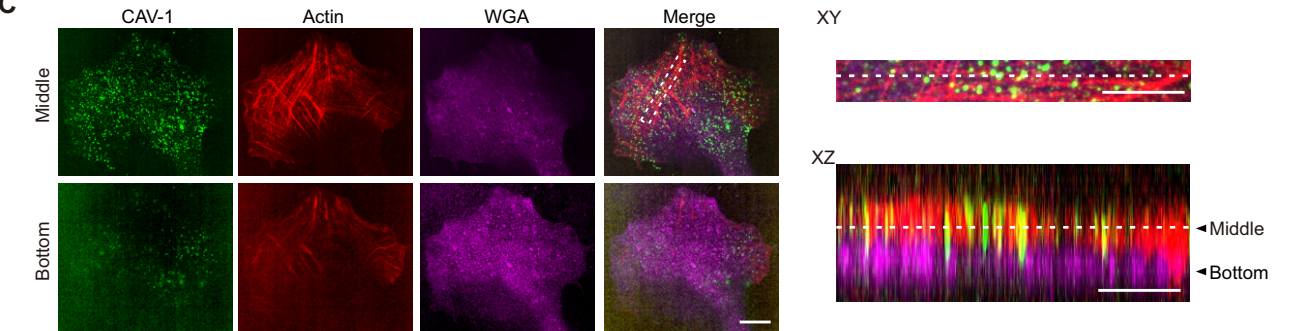

D

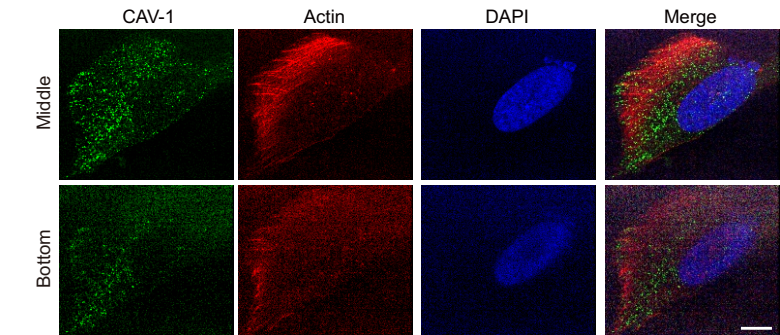

Figure S2

A

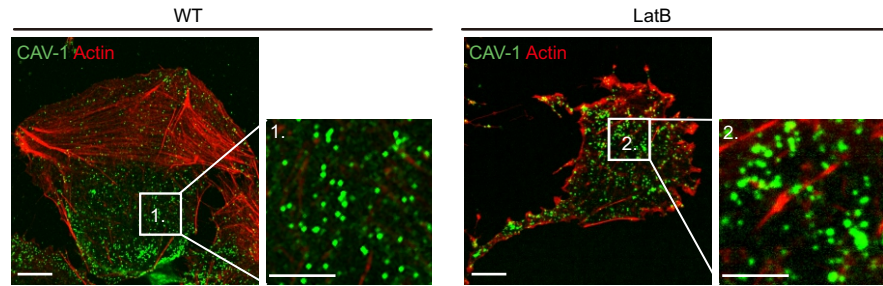

B

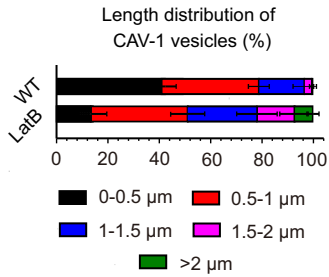

C

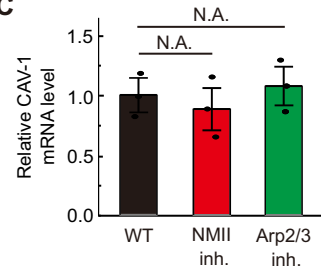

D

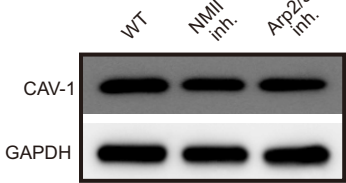

E

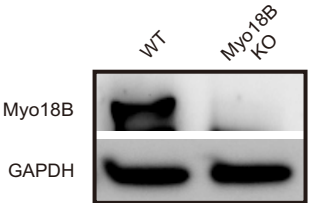

F

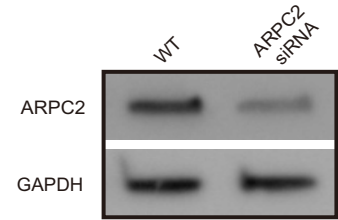

G

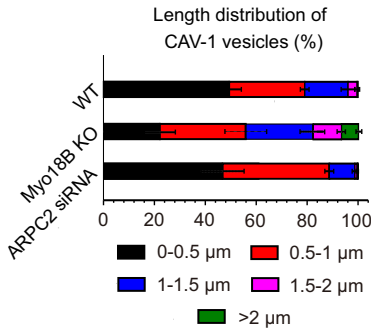

H

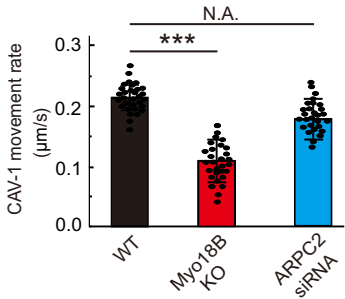

**Figure S3****A**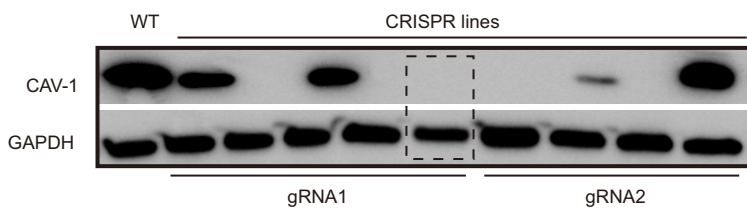**B**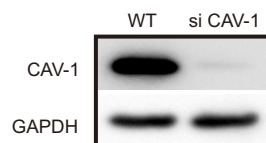**C**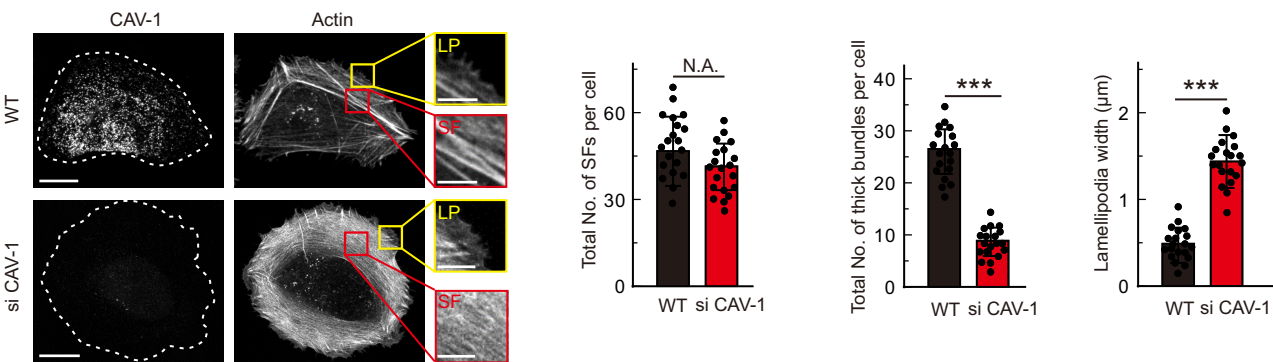**D**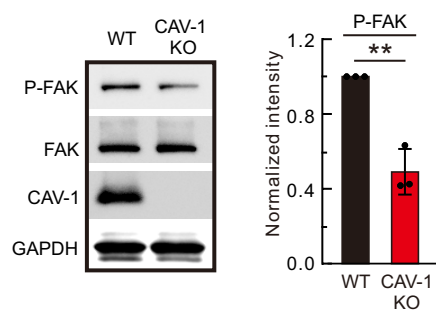**E**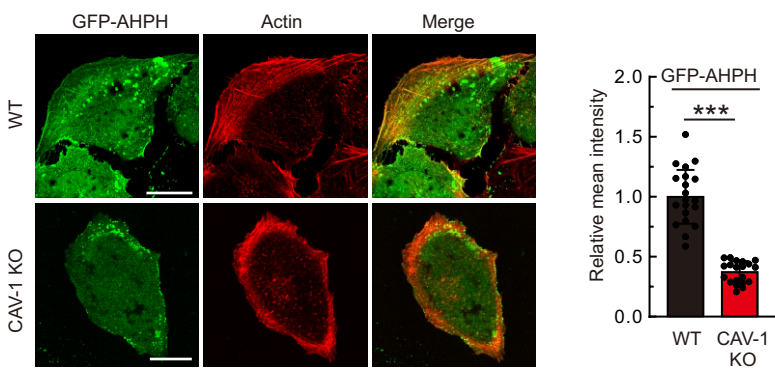**F**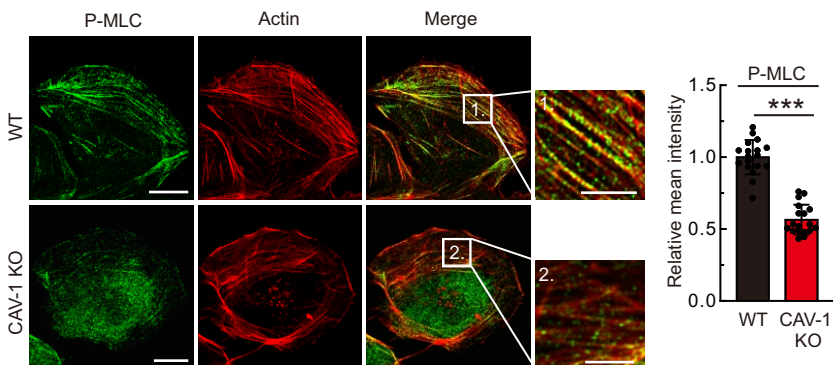**G**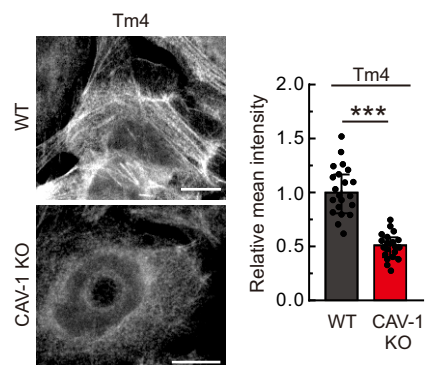

Figure S4

A

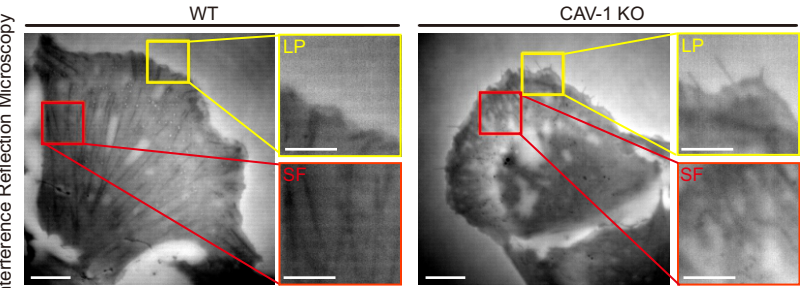

B

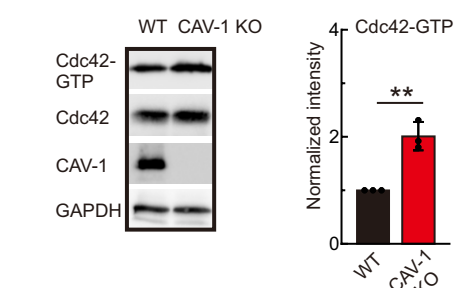

C

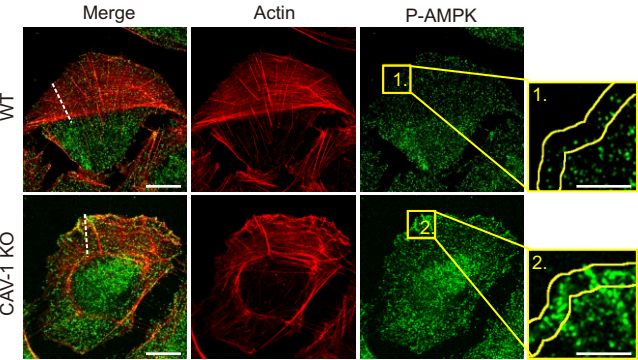

D

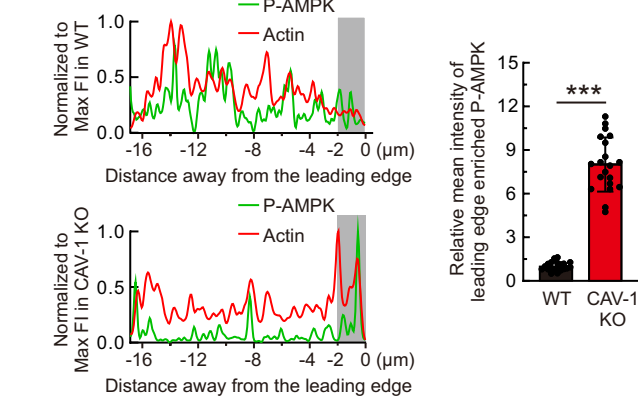

E

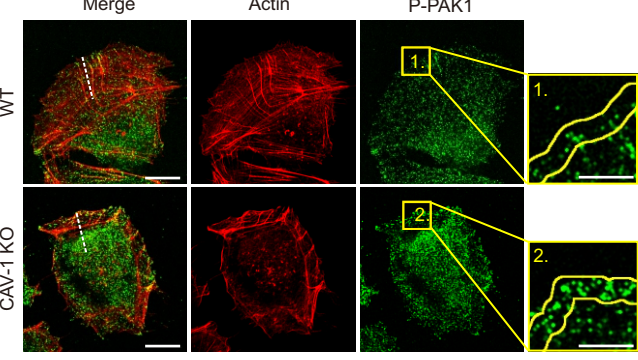

F

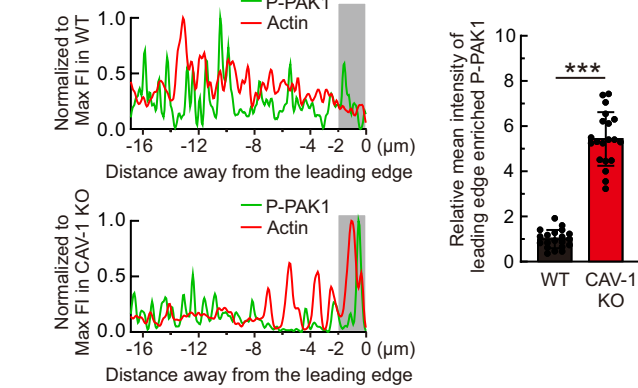

Figure S5

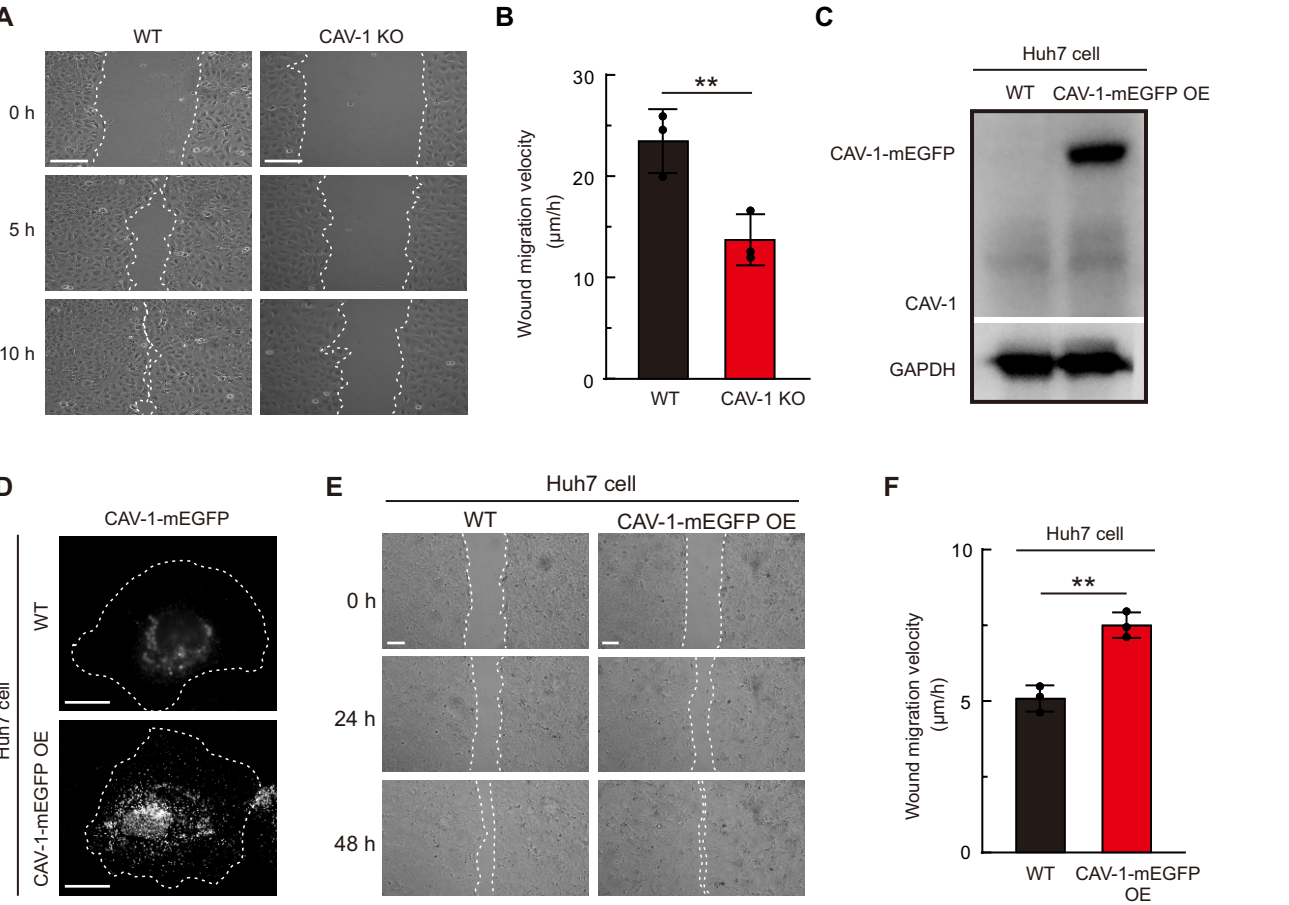

Supplement: Supplementary Figure 1 — The expression and motility of CAV-1 vesicles in U2OS cells. (A) A schematic cartoon demonstrates that tropomyosin is a tightly actin associated components. Immunofluorescence staining of Biotin fused Tpm3.1 and streptavidin localized to actin filaments to verify the screen are shown in the lower panel. Bars, 10 μm. (B) Representative immunofluorescence images of overexpressing CAV-1-mCherry paired with endogenous CAV-1 and Rab8 antibodies, respectively. The yellow box in the merged images show magnified images. Bars, 10 μm (in cell images) and 5 μm (in the magnified box). (C) Localization of endogenous CAV-1 and actin filaments in U2OS cells detected by CAV-1 antibody and fluorescent phalloidin, respectively. WGA was used to label the plasma membrane. Magnified regions from the area indicated by white boxes demonstrate that cytoplasmic CAV-1 aligns with actin filaments. The orthogonal view shows the view of the enlarged region, where “XY” and “XZ” indicated different cross-sections. (D) Representative images of actin filaments and endogenous CAV-1. DAPI was used to mark the nucleus. “Middle” refers to the cytoplasmic field of the cell, and “Bottom” indicates the ventral plasma membrane area. Bars, 10 μm (in cell images), 5 μm (in magnified images and orthographic views). [file Data_Sheet_1.PDF]
